# A novel gene’s role in an ancient mechanism: secreted Frizzled-related protein 1 is a critical component in the anterior–posterior Wnt signaling network that governs the establishment of the anterior neuroectoderm in sea urchin embryos

**DOI:** 10.1186/s13227-017-0089-3

**Published:** 2018-01-22

**Authors:** Anita Khadka, Marina Martínez-Bartolomé, Stephanie D. Burr, Ryan C. Range

**Affiliations:** 1 Department of Biological Sciences, Mississippi State University, Mississippi State, MS 39762 USA; 2 School of Pharmacy, University of Mississippi, Oxford, MS USA

**Keywords:** Developmental biology, Neuroectoderm patterning, Wnt signal transduction, Anterior–posterior, Deuterostome evolution, Gene regulatory networks, Dkk1, sFRP-1/2/5, Fzl1/2/7, Fzl5/8

## Abstract

The anterior neuroectoderm (ANE) in many deuterostome embryos (echinoderms, hemichordates, urochordates, cephalochordates, and vertebrates) is progressively restricted along the anterior–posterior axis to a domain around the anterior pole. In the sea urchin embryo, three integrated Wnt signaling branches (Wnt/β-catenin, Wnt/JNK, and Wnt/PKC) govern this progressive restriction process, which begins around the 32- to 60-cell stage and terminates by the early gastrula stage. We previously have established that several secreted Wnt modulators of the Dickkopf and secreted Frizzled-related protein families (Dkk1, Dkk3, and sFRP-1/5) are expressed within the ANE and play important roles in modulating the Wnt signaling network during this process. In this study, we use morpholino and dominant-negative interference approaches to characterize the function of a novel Frizzled-related protein, secreted Frizzled-related protein 1 (sFRP-1), during ANE restriction. sFRP-1 appears to be related to a secreted Wnt modulator, sFRP3/4, that is essential to block Wnt signaling and establish the ANE in vertebrates. Here, we show that the sea urchin sFRP3/4 orthologue is not expressed during ANE restriction in the sea urchin embryo. Instead, our results indicate that ubiquitously expressed maternal sFRP-1 and Fzl1/2/7 signaling act together as early as the 32- to 60-cell stage to antagonize the ANE restriction mechanism mediated by Wnt/β-catenin and Wnt/JNK signaling. Then, starting from the blastula stage, Fzl5/8 signaling activates zygotic sFRP-1 within the ANE territory, where it works with the secreted Wnt antagonist Dkk1 (also activated by Fzl5/8 signaling) to antagonize Wnt1/Wnt8–Fzl5/8–JNK signaling in a negative feedback mechanism that defines the outer ANE territory boundary. Together, these data indicate that maternal and zygotic sFRP-1 protects the ANE territory by antagonizing the Wnt1/Wnt8–Fzl5/8–JNK signaling pathway throughout ANE restriction, providing precise spatiotemporal control of the mechanism responsible for the establishment of the ANE territory around the anterior pole of the sea urchin embryo.

**Electronic supplementary material:**

The online version of this article (10.1186/s13227-017-0089-3) contains supplementary material, which is available to authorized users.

## Background

Metazoan embryos share a remarkably conserved toolkit of signal transduction pathways and transcription factors, which often are arranged into conserved gene regulatory networks (GRNs) that drive similar developmental processes. Conversely, these conserved toolkit components can also be re-arranged into novel GRNs that lead to morphological differences among species [1, 2]. While most evolutionary and developmental biology studies focus on these conserved toolkit genes, relatively few functional studies are performed on the novel genes that comprise a significant proportion of sequenced genomes [3, 4]. These novel genes can be a powerful mechanism for generating morphological diversity among animals, and most studies have been focused on this aspect [3, 5]. However, another important question that has been largely overlooked is what happens when novel gene products are incorporated into the more ancient signal transduction pathways and transcriptional regulatory networks that drive fundamental development processes and maintain adult tissue homeostasis in a variety of animals.

The molecular components involved in Wnt signaling are highly conserved in metazoans from cnidarians to humans. Three main branches have been identified: the “canonical” Wnt/β-catenin pathway and the “alternative” Wnt/JNK and Wnt/Ca^2+^ pathways. Each of these pathways uses a combination of secreted Wnt ligands and transmembrane Frizzled receptors as well as various co-receptors and secreted modulators to influence the activity of its respective intracellular signaling pathway. The specific developmental roles for Wnt signaling are also often remarkably conserved among many metazoan embryos. One of these is the gradient of Wnt signaling that is essential for territory patterning along the primary embryonic axis (anterior–posterior, oral–aboral, animal–vegetal). In most metazoans, high Wnt signaling specifies endoderm around the end of the primary axis where the blastopore will form, while low Wnt signaling levels allow for ectodermally derived sensory organ specification on the opposite side of the embryo [6–14]. In many deuterostome embryos (echinoderms, hemichordates, urochordates, cephalochordates, vertebrates), patterning along the primary anterior–posterior (AP) axis can be observed by the gradual restriction of the anterior neuroectoderm (ANE) gene regulatory network (GRN) activity to a region around the anterior pole of the embryo (for review see [28]). In deuterostome embryos in which AP neuroectoderm patterning has been studied, early ANE GRN members, such as Six3, initially are expressed broadly throughout the presumptive neuroectoderm territory [7, 15–19]. Then, a mechanism that depends on the posterior-to-anterior gradient of Wnt/β-catenin signaling progressively eliminates ANE GRN expression from more posterior ectoderm, ultimately confining it to a territory around the anterior pole where secreted Wnt antagonists block Wnt signaling [7, 19–23]. Once restricted around the anterior pole, these ANE territories subsequently give rise to structures ranging from the simple anterior sensory organs of invertebrate deuterostomes to the more complex vertebrate forebrain and eye fields [24–29]. Importantly, comparative gene expression and functional studies among deuterostome embryos reveal significant similarities in the gene regulatory networks (GRNs) governing the specification and patterning of the territories from which anterior neurogenic organs arise [28], suggesting that they may be homologous.

Previous studies have shown that the ANE restriction mechanism in sea urchin embryos begins to downregulate ANE gene expression as early as the 32-cell stage when high Wnt/β-catenin signaling represses the expression of two early cardinal regulators of the ANE GRN, Six3 and FoxQ2, in the posterior half of the embryo, restricting their expression to the anterior hemisphere [19, 21]. In addition to antagonizing ANE GRN expression and activating endomesoderm specification, Wnt/β-catenin signaling also activates the expression of two secreted Wnt ligands, Wnt1 and Wnt8, in posterior endomesodermal blastomeres at the 32-cell stage. These ligands appear to diffuse to more anterior blastomeres, where they activate a Wnt/JNK signaling pathway through the Fzl5/8 receptor, which acts as a relay to further downregulate the expression of the ANE GRN in the ectoderm territory. Simultaneously, Fzl1/2/7–PKC signaling antagonizes Wnt/JNK signaling in ectodermal cells, preventing the elimination of ANE GRN expression from anterior-most cells during the initial and middle stages of ANE positioning. During the final phase of the process, Fzl5/8 signaling activates the expression of a secreted Wnt antagonist, Dkk1, in the cells around the anterior pole. Dkk1 prevents the downregulation of ANE factors within this territory by antagonizing Fzl5/8 signaling through a negative feedback mechanism [20], establishing a correctly sized ANE territory. As a result of this process, three early domains are established along the AP axis: endomesoderm around the posterior end; an equatorial ectodermal band that will form ventral and dorsal ectodermal structures separated by the ciliary band and associated nerves; and the anterior neuroectoderm domain around the anterior pole. These observations established that integrated information from all three of these Wnt signaling pathways patterns cell fates along the anterior–posterior axis in sea urchins, and we propose that these form a Wnt signaling network [20]. Remarkably, comparison of functional and expression studies among deuterostome embryos strongly suggests that aspects of this mechanism uncovered in the sea urchin embryo may be conserved among deuterostome embryos [28].

**Fig. 1:**
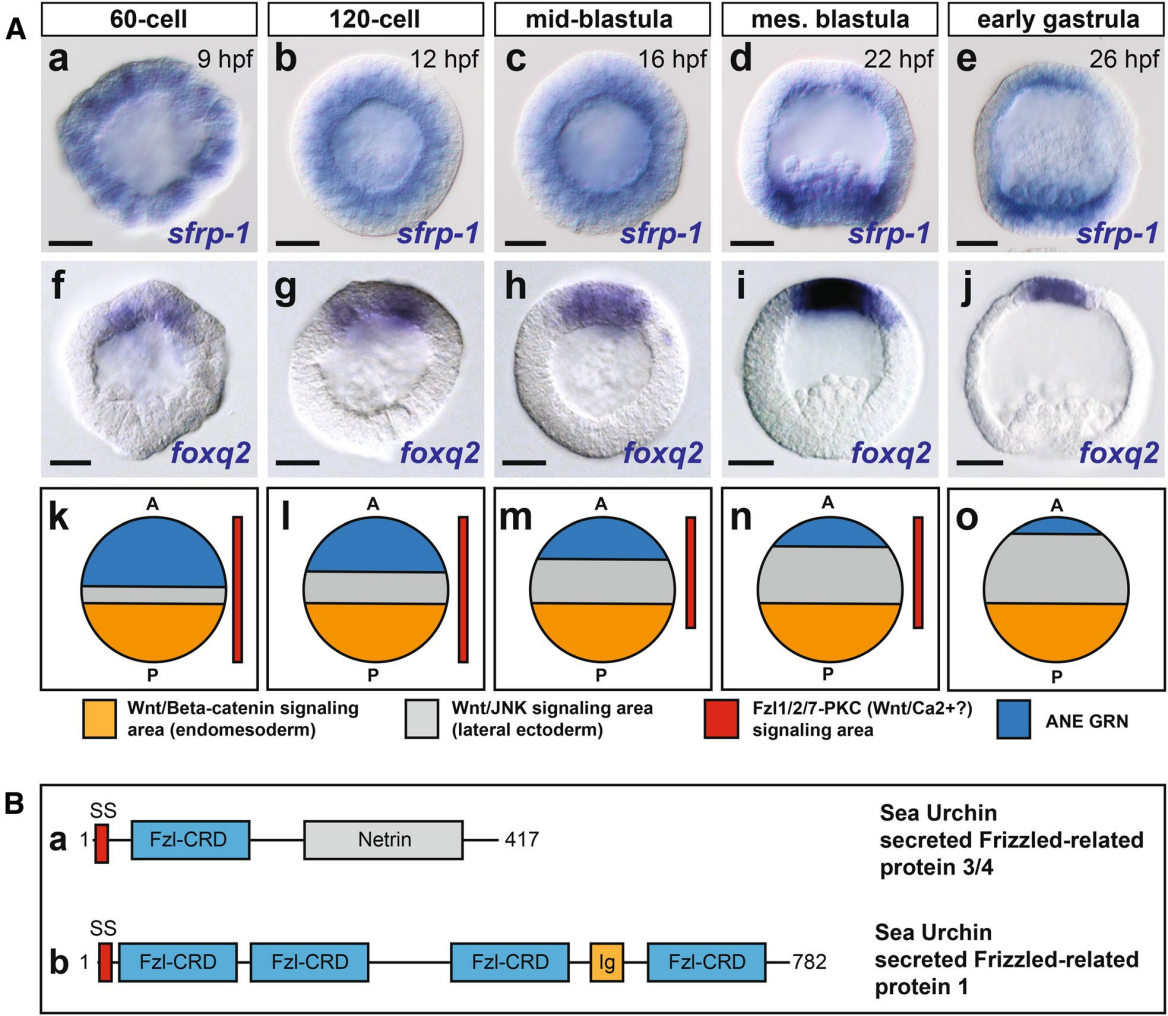
Spatiotemporal expression of sFRP-1 during ANE restriction. **Aa**–**c** *sfrp*-*1* expression is first detected at the 32- to 60-cell stage and is present throughout the embryo until mid-blastula stage. **Ad**, **Ae** Between mesenchyme blastula stage and early gastrula, *sfrp*-*1* is restricted to anterior and posterior blastomeres. **Af**–**j** *foxq2* expression is first detected at 32- to 60-cells stage in the anterior blastomeres and is progressively downregulated from posterior ectoderm cells from the 60-cell to mesenchyme blastula stages as previously shown [21]. **Ak**–**o** Diagram showing a model for the areas of Wnt/β-catenin, Fzl5/8–JNK, and Fzl1/2/7–PKC signaling during ANE restriction consistent with the data from [20]. Each individual diagram corresponds to the developmental stages depicted in the panels directly above. **B** Functional protein domain organization for the CRD containing proteins including sea urchin sFRP3/4 (**a**) and secreted Frizzled-related protein 1 (**b**). Scale bar = 20 μm

While much is known about many of the components of the individual signaling branches, relatively little is known about the specific molecular mechanisms that mediate coordinated interactions among them in any in vivo developmental model. Secreted extracellular Wnt modulators (e.g., sFRPs and Dkks) are obvious candidates for mediating these interactions during AP neuroectoderm patterning, since they have been shown to play essential roles during primary axis patterning in several metazoan embryos [20, 30–32], including those of sea urchins and chordates. Phylogenetic analyses suggest that the eumetazoan ancestor possessed six *Frizzled*-*related* genes that included four Frizzled receptors (*fzl1/2/7, fzl4, fzl5/8, fzl9/10*) and two secreted Frizzled-related genes (*sfrp-1/2/5* and *sfrp3/4*) [33, 34]. The sea urchin genome contains the full Frizzled-related complement, and all of these genes are expressed during early embryonic development except for *sfrp3/4* [35, 36]. Because vertebrate sFRP3/4 orthologues (FrzBs) play critical roles in early AP patterning and anterior specification processes in vertebrates [37–39], the silence of *sfrp3/4* is curious. The core domain that each of these Frizzled-related genes contain is the Frizzled cysteine-rich domain (Fzl-CRD), which is critical for Wnt ligand interactions [40, 41]. Interestingly, the sea urchin also possesses a set of 15 novel Fzl-CRD-containing proteins of unknown function (see Additional file 1: Figure S1A) [34]. In 2002, Illies et al. [43] described the domain architecture and spatiotemporal expression of one of these novel Fzl-CRD-containing proteins; they termed secreted Frizzled-related protein 1 (sFRP-1) in early *Strongylocentrotus purpuratus* embryos that appears to be related to the ancient lineage of secreted Frizzled-related protein 3/4 (sFRP3/4) [35]. Similar to other sFRPs, this protein contains a signal sequence and at least one cysteine-rich domain (CRD), while lacking transmembrane domains. However, unlike sFRPs, this novel protein does not possess a Netrin domain [30, 42], but instead contains 4 tandem CRD domains with a single Ig domain situated in between CRD3 and CRD4 (see Fig. 1Aa). These four CRDs are well conserved with one another and with the CRDs of sFRPs and Frizzled receptors, maintaining the highly conserved spacing of the 10 characteristic cysteine residues. *sfrp*-*1* is ubiquitously expressed during early blastula stages and then prominently expressed in both the endoderm and the ANE territory by mesenchyme blastula/early gastrula stages [43], overlapping spatiotemporally with the region in which the Wnt network is controlling the establishment ANE GRN around the anterior pole.

Here we present the first functional study of the novel secreted Frizzled-related protein 1 gene. Our data indicate that ubiquitously expressed, maternal sFRP-1 function is necessary, along with Fzl1/2/7 signaling, to antagonize the early ANE restriction mechanism mediated by Wnt/β-catenin and Wnt/JNK signaling. In addition, we found that Fzl5/8–JNK signaling activates zygotic expression of sFRP-1 in anterior cells during the later stages of ANE restriction, where sFRP-1 acts together with secreted Dkk1 to antagonize Fzl5/8–JNK signaling through a negative feedback mechanism to establish the ANE territory.

## Results

### *sfrp*-*1* and *sfrp3/4* expression during ANE restriction

The sea urchin genome contains at least 15 novel Fzl-CRD-containing genes [34, 44]. A phylogenetic analysis of the individual Fzl-CRD domains from these proteins indicates these proteins form six groups (Additional file 1: Figure S1A). In this study, we focus on a novel secreted Frizzled-related gene, termed sFRP-1 [43], that clusters with the Fzl-CRD domains of ancestral Frizzled receptors and sFRPs (Additional file 1: Figure S1A). Previously, Illies et al. [43] showed that *sfrp*-*1* transcripts are expressed in a spatiotemporal profile consistent with a role in early AP patterning. Using northern blot and in situ hybridization approaches, they found that *sfrp*-*1* transcripts were not expressed until early blastula stage. Using more sensitive qPCR analyses, we detected *sfrp*-*1* transcripts in the fertilized egg and at the 60-cell stage (9 hpf) (see Fig. 5Ab). We were also able to detect ubiquitously expressed *sfrp*-*1* transcripts by whole-mount in situ hybridization as early as the 60-cell stage (Fig. 1Aa). Consistent with the earlier report [43], high levels of *sfrp*-*1* expression were detected during early to mid-blastula stages, when these transcripts were expressed broadly throughout the embryo (Fig. 1Ab, Ac). Subsequently, *sfrp*-*1* expression was downregulated in the central ectoderm territory during mid-to-late blastula stages (Fig. 1Ad), resulting in localized expression in the ANE and posterior endomesoderm territories by the mesenchyme blastula/early gastrula stage (Fig. 1Ae). As a measure of ANE restriction, we assayed the downregulation of expression of the cardinal ANE regulatory factor *foxq2* from lateral ectodermal cells, which initiates around 60-cell stage and terminates at mesenchyme blastula stage (24 hpf) (Fig. 1Af–j). These results indicate that *sfrp*-*1* is expressed in all ectoderm cells in which ANE gene expression is downregulated by Wnt1/Wnt8–Fzl5/8–JNK signaling during the initial stages of ANE restriction. Then, *sfrp*-*1* is restricted to the ANE territory around the same time that ANE restriction terminates [20]. In addition, the expression of *sfrp*-*1* in the posterior endomesoderm cells during the mesenchyme blastula/early gastrula stage (Fig. 1Ae) suggests that sFRP-1 is also involved in the specification of this territory and/or the morphogenetic movements during gastrulation.

sFRP-1’s Fzl-CRD domains are related to those of sFRP3/4, suggesting that sFRP-1 may have emerged from the ancestral sFRP3/4 (Fig. 1B) [35]. Interestingly, tiling and transcriptome data suggest that the ancestral sea urchin *sfrp3/4* gene is not transcribed during early sea urchin development [35]. To confirm these data, we performed qPCR analysis on embryos at several developmental time points during ANE restriction, which showed that only 1–4 *sfrp3/4* transcripts *per embryo* were expressed at any developmental stage during ANE restriction (Additional file 1: Figure S1B), suggesting that in contrast to vertebrate embryos the ancestral sFRP3/4 is not necessary for early anterior–posterior development in sea urchin embryos.

### sFRP-1 is necessary for ANE territory specification

**Fig. 2:**
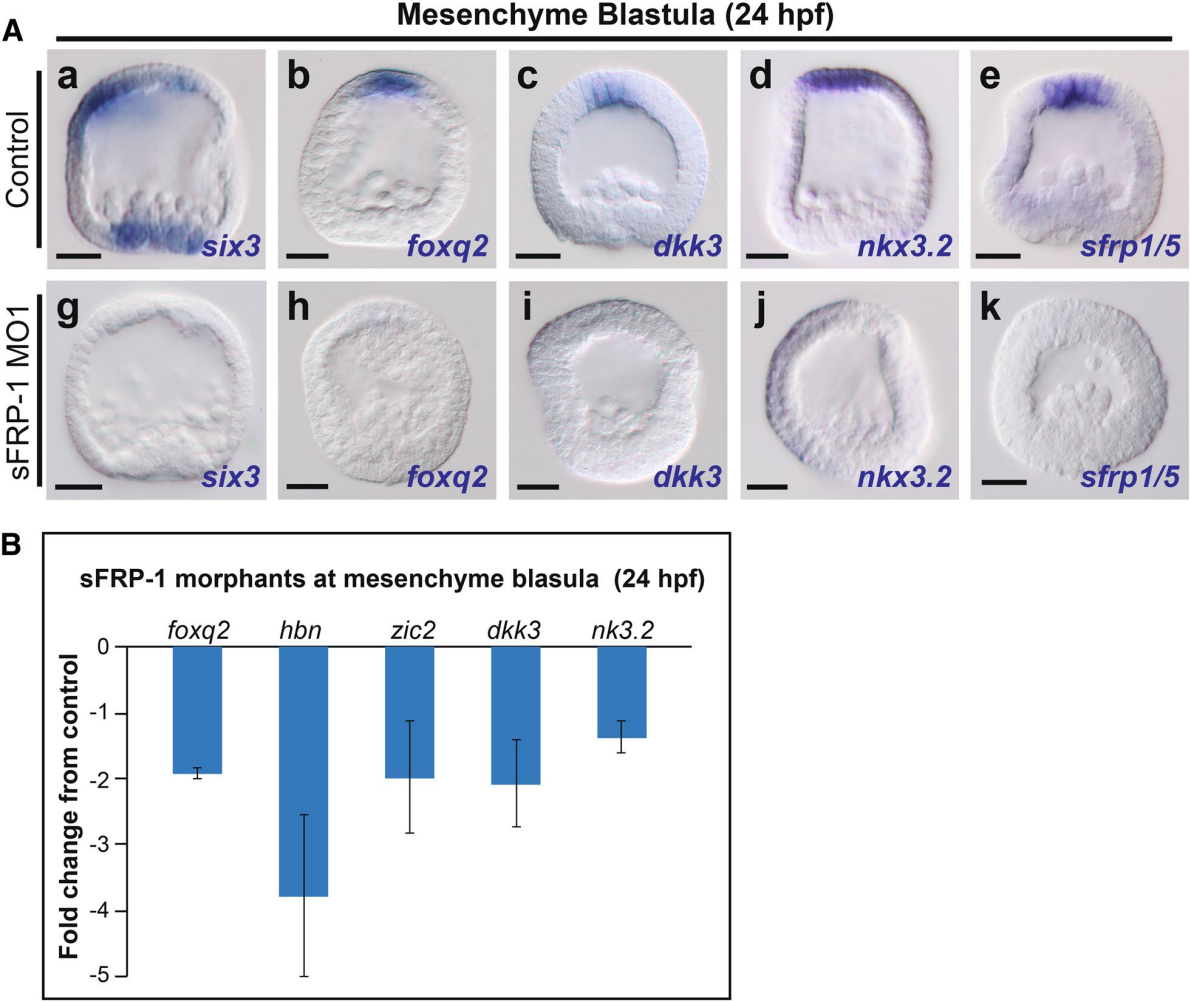
sFRP-1 is necessary for ANE specification. **Aa**–**e** *six3, foxq2*, *dkk3*, *nkx3.2*, and *sfrp-1/5* expression in mesenchyme blastula stage control embryos (24 hpf). **Ag**–**k** Embryos injected with sFRP-1 morpholino 1 show complete elimination of ANE markers. **B** qPCR measurements from three different cultures of embryos showing the downregulation of ANE regulatory genes at mesenchyme blastula stage (24hpf) in sFRP-1 morphants. The *y* axis shows the fold change in gene expression for sFRP-1 morphants versus controls. Scale bar = 20 μm. MO, morpholino

The domain structure and spatial expression of *sfrp*-*1* suggest that it could function to modulate Wnt signaling during ANE restriction. To test this hypothesis, we performed knockdowns with two different morpholino oligonucleotides and analyzed ANE GRN expression at mesenchyme blastula stage (24 hpf) (Fig. 2; Additional file 2: Figure S2). Whole-mount in situ hybridization (WMISH) showed that the cardinal ANE regulators *six3* and *foxq2,* as well as downstream ANE GRN components *dkk3*, *nkx3.2*, *sfrp-1/5* were downregulated in sFRP-1 morphants (Fig. 2A; Additional file 2: Fig. 2A). In addition, qPCR analysis showed that the expression of *foxq2, nkx3.2,* and *dkk3* as well as that of two other components of the ANE GRN, *hbn* and *zic2*, was consistently downregulated in sFRP-1 morphants at the mesenchyme blastula stage (24 hpf) (Fig. 2B). These results show that sFRP-1 is necessary for the specification of the ANE territory, possibly through a role in antagonizing the ANE restriction mechanism mediated by Wnt/β-catenin and Wnt1/Wnt8/Fzl5/8–JNK signaling.

### sFRP-1 antagonizes Fzl5/8 signaling throughout ANE restriction

**Fig. 3:**
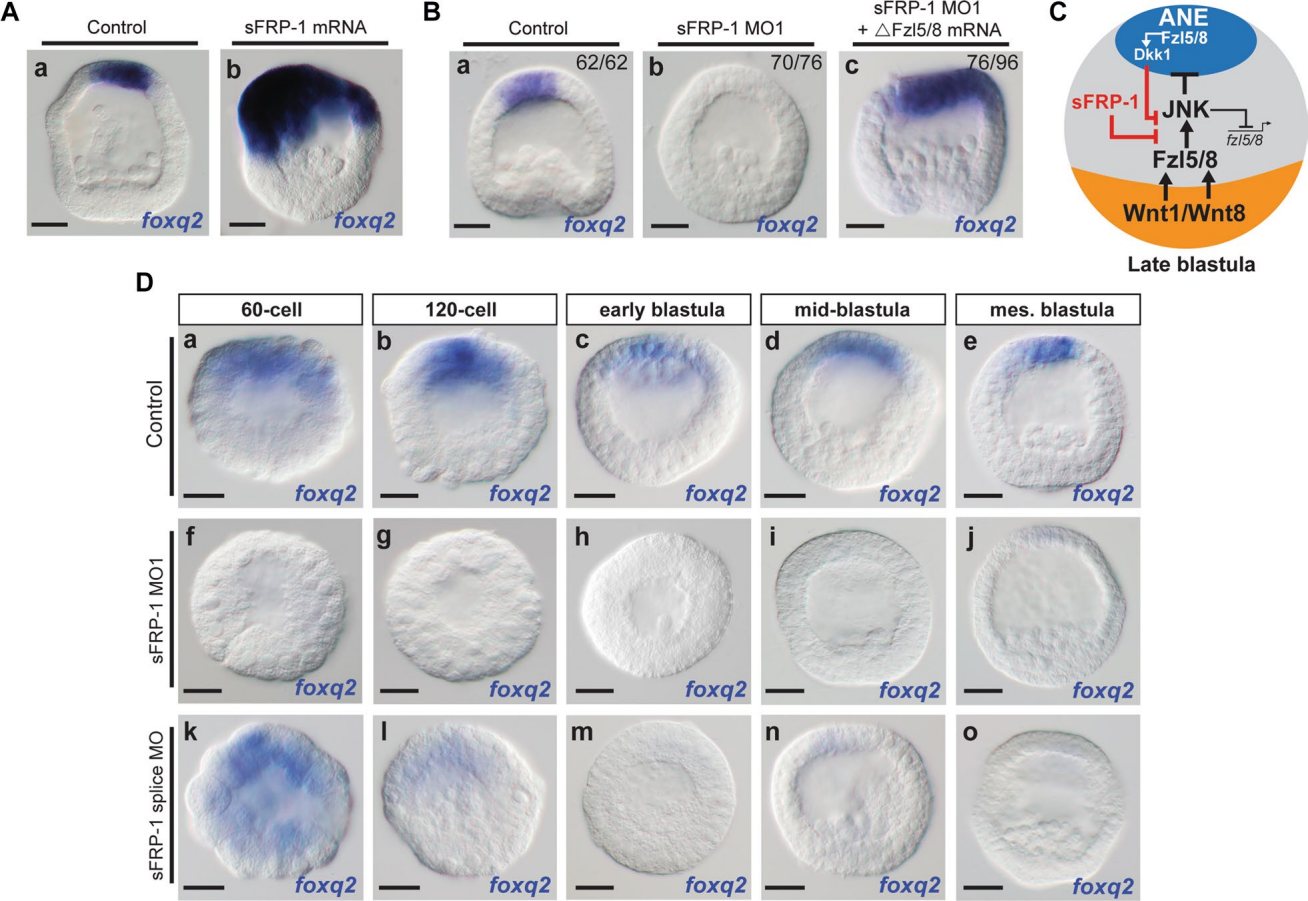
Function of maternal and zygotic sFRP-1 during ANE restriction. **A** Compared to control embryos (**a**), embryos injected with *sfrp*-*1* mRNA show expanded *foxq2* expression (**b**). **B** In the absence of sFRP-1, *foxq2* expression is completely eliminated from ANE territory (**Bb**), whereas sFRP-1 morphants co-injected with ΔFzl5/8 show expanded *foxq2* expression (**Bc**). The number of embryos examined that show the representative phenotypes depicted is indicated in each panel. **C** A model diagram for ANE restriction during late blastula stages showing Wnt1/Wnt8–Fzl5/8–JNK signaling downregulates the transcription of *fzl5/8* in posterior ectoderm, restricting its expression to the ANE territory. In the ANE, Fzl5/8 signaling activates *dkk1* expression, antagonizing Wnt1/Wnt8–Fzl5/8–JNK signaling and establishing the ANE territory. Maternal and/or zygotic sFRP-1 is also necessary to antagonize Fzl5/8 signaling at this stage of development. The model is based data presented in this figure and from [20]. **D** Expression of *foxq2* from 60-cell stage to mesenchyme blastula stage in control embryos (**Da**–**e**), in translation-blocking sFRP-1 morphants (**Df**–**j**) and splice-blocking sFRP-1 morphants (**Dk**–**o**). MO, morpholino. Scale bar = 20 μm

We previously identified two components (Dkk1 and Fzl1/2/7 signaling) of the molecular mechanism(s) necessary to antagonize Wnt1/Wnt8–Fzl5/8–JNK signaling emanating from posterior/vegetal blastomeres during ANE restriction. If each of these components is knocked down individually, Fzl5/8–JNK signaling appears to be upregulated, resulting in complete elimination of ANE GRN expression. Importantly, the expression of the ANE GRN can be rescued in Dkk1 and Fzl1/2/7 knockdown embryos if Fzl5/8 signaling is inhibited [20, 31]. Together, these results indicate that both Fzl1/2/7 signaling and Dkk1 are essential to establish the ANE territory. Based on the sFRP-1 morpholino knockdown phenotype, we reasoned that secreted sFRP-1 may also function to protect the ANE GRN by antagonizing Fzl5/8–JNK signaling. To begin to test this idea, we injected zygotes with synthetic *sfrp*-*1* mRNA and found that expression of *foxq2* expanded toward the posterior/vegetal pole at mesenchyme blastula stage (24 hpf) (Fig. 3A), suggesting that high levels of sFRP-1 interfere with Fzl5/8’s downregulation of the ANE GRN. Next, within each of the three batches of zygotes we injected one set with sFRP-1 MO1 and a second set with sFRP-1 MO1 and mRNA encoding a dominant-negative form of the Fzl5/8 receptor (ΔFzl5/8). As before, *foxq2* expression was significantly downregulated or completely undetectable in embryos lacking sFRP-1 (Fig. 3Bb). In contrast, a large majority of fertilized embryos injected with sFRP-1 MO1 and ΔFzl5/8 mRNA showed expanded *foxq2* expression (Fig. 3Bc), indicating that, in the absence of functional Fzl5/8, the ANE GRN is still activated in sFRP-1 morphants. Taken together, these results indicate that sFRP-1 is necessary to antagonize Fzl5/8–JNK signaling and therefore inhibit the downregulation of the ANE GRN (Fig. 3C).

The spatial and temporal expression patterns of *sfrp*-*1* suggest it may function throughout the period of ANE restriction (32/60-cell stage to mesenchyme blastula). To test this hypothesis, we used separately a translation-blocking morpholino (sFRP-1 MO1) to knock down the function of both maternally and zygotically synthesized sFRP-1 mRNA and a splice-blocking morpholino (sFRP-1 MO2) to knock down only zygotic sFRP-1 activity and assayed for *foxq2* expression. In translation-blocking morphants, *foxq2* expression was completely downregulated in 60-cell stage embryos and downregulation was maintained until mesenchyme blastula stage (Fig. 3Df–j). In splice-blocking morphants, *foxq2* expression was detected in 60-cell stage and 120-cell stage embryos (Fig. 3Dk, Dl) and then downregulated in early blastula stage and mesenchyme blastula stage embryos (Dm–o). Together, these results indicate that maternal and/or early zygotic sFRP-1 transcripts are necessary to antagonize Fzl5/8–JNK-mediated downregulation of ANE GRN from the beginning of the restriction process. Then, during the blastula stages, zygotic sFRP-1 activity is necessary to insulate the ANE territory by antagonizing Fzl5/8–JNK signaling.

### sFRP-1 and Fzl1/2/7 signaling act together, but independently to antagonize ANE restriction

**Fig. 4:**
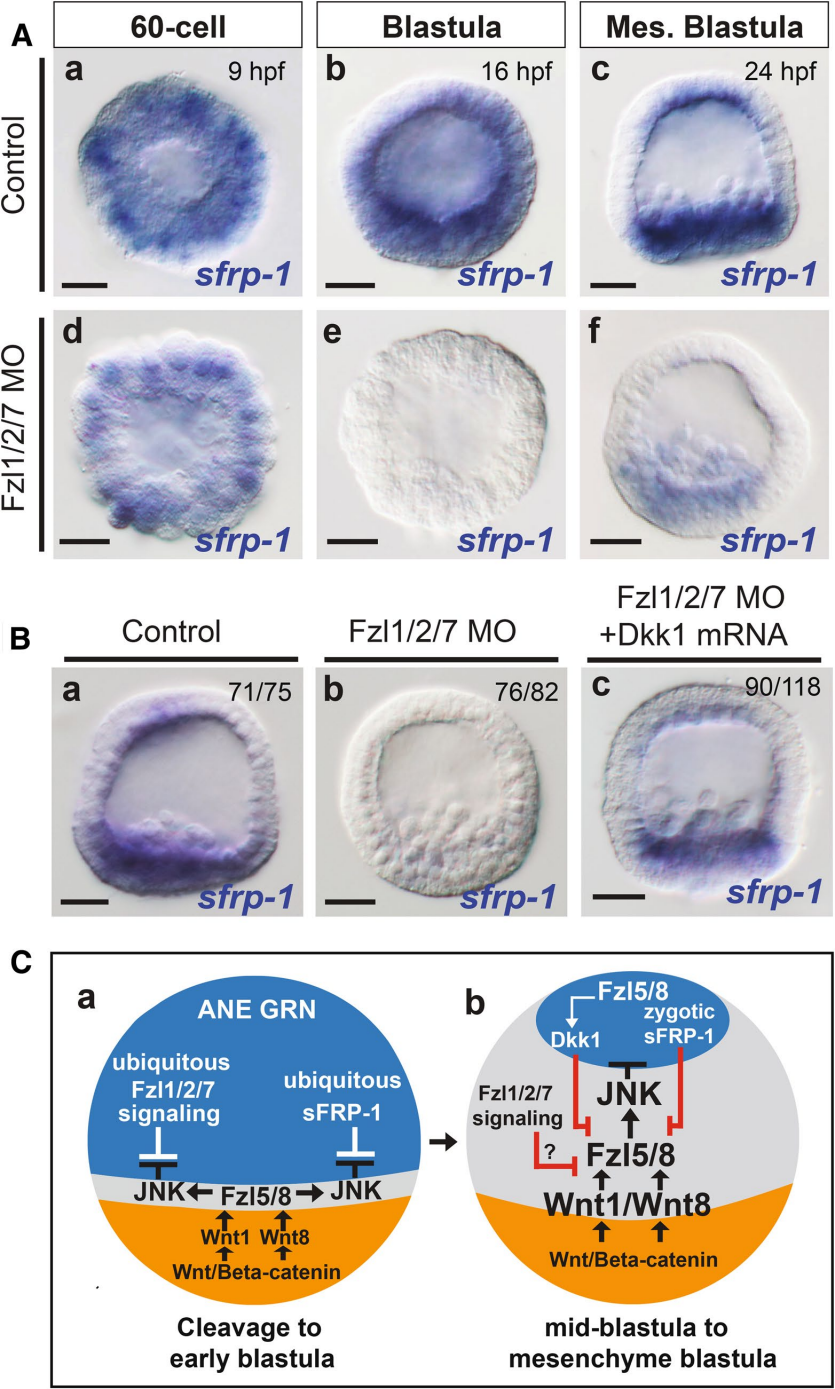
Control of *sfrp*-*1* expression by the Wnt signaling network governing ANE restriction. **A** Fzl5/8–JNK signal-mediated downregulation of ANE gene expression is enhanced in the absence of Fzl1/2/7. Control embryos showing *sfrp*-*1* expression at 60-cell (**a**), blastula (**b**) and mesenchyme blastula stage (**c**). **Ad**–**f** In Fzl1/2/7 MO injected embryos, *sfrp*-*1* is expressed at the 60-cell stage (**d**), but downregulated in blastula (**e**) and mesenchyme blastula embryos (**f**). **Ba**–**c** *sfrp*-*1* expression in Fzl1/2/7 morphants is rescued by overexpressing *dkk1* mRNA. The number of embryos examined that show the representative phenotypes depicted is indicated in each panel. **C** A model diagram, showing that maternal sFRP-1 works together with, but independently from, Fzl1/2/7 signaling during the early stages of ANE restriction (**Ca**) and that zygotic sfrp-1 is part of the ANE GRN downregulated by Wnt/JNK signaling during later stages of the process (**Cb**). The model also shows that during later stages of ANE restriction the Fzl5/8 receptor is a member of the ANE GRN and Fzl5/8–JNK signaling downregulates fzl5/8 expression in the lateral ectoderm. The model is based on the data presented in this and the preceding figures, as well as data from [20]. MO, morpholino. Scale bar = 20 μm

In a previous study, we showed that Fzl1/2/7–PKC signaling is necessary to antagonize the downregulation of the ANE GRN by Wnt/β-catenin and Fzl5/8–JNK signaling from as early as the 32- to 60-cell stage at the beginning of ANE restriction [20]. Above we showed that *foxq2* expression was eliminated in sFRP-1 morphants during early cleavage stages (Fig. 3Df), mimicking the phenotype we observed when Fzl1/2/7–PKC signaling was blocked. Thus, it is possible that sFRP-1 plays a role either downstream of, or in parallel with, Fzl1/2/7 to antagonize the restriction process. To determine the relationship between sFRP-1 and Fzl1/2/7 signaling, we injected zygotes with Fzl1/2/7 morpholino oligonucleotides and observed expression of *sfrp*-*1* in the embryos at multiple stages during ANE restriction. At the 60-cell stage, *sfrp*-*1* was still expressed in the absence of Fzl1/2/7 signaling (cf. Figure 4Aa, Ad). However, starting from mid-blastula stage, *sfrp*-*1* expression initially was lost in the whole embryo and the loss persisted in the anterior hemisphere throughout ANE restriction, although low levels of expression were reactivated in the posterior endomesoderm territory (Fig. 4Ae, Af). These data indicate that Fzl1/2/7 signaling is not necessary for *sfrp*-*1* expression during early cleavage stages, but it might be necessary for its later zygotic expression in the anterior and posterior territories of the embryo. In the absence of Fzl1/2/7 signaling, Fzl5/8–JNK signaling is unchecked in the anterior half of the embryo; thus, Fzl5/8–JNK signaling in anterior blastomeres in the absence of Fzl1/2/7–PKC antagonism could alternatively be responsible for the downregulation of *sfrp*-*1* in Fzl1/2/7 morphants. To distinguish between these alternatives, we compared *sfrp*-*1* expression in embryos injected Fzl1/2/7 MO alone and those injected with Fzl1/2/7 MO and high levels of synthetic *dkk1* mRNA (Fig. 4B). We have previously shown that Dkk1 acts as a secreted Wnt modulator that antagonizes the Wnt/JNK signaling-mediated downregulation of the ANE GRN [31]. *sfrp*-*1* expression persisted in the embryos injected with Fzl1/2/7 MO along with *dkk1* mRNA, while it was eliminated in the embryos injected with Fzl1/2/7 MO alone (Fig. 4Bb, Bc), indicating that in the absence of Fzl1/2/7 signaling, Fzl5/8 signaling downregulates *sfrp*-*1* during the later stages of ANE restriction. Together, these observations indicate that maternal sFRP-1 works together with, but independently from, Fzl1/2/7–PKC signaling to antagonize Wnt/β-catenin and/or Wnt/JNK signaling that drive downregulation of the ANE GRN (Fig. 4C).

### sFRP-1 acts in parallel with Dkk1 downstream of Fzl5/8 signaling to establish the ANE territory

**Fig. 5:**
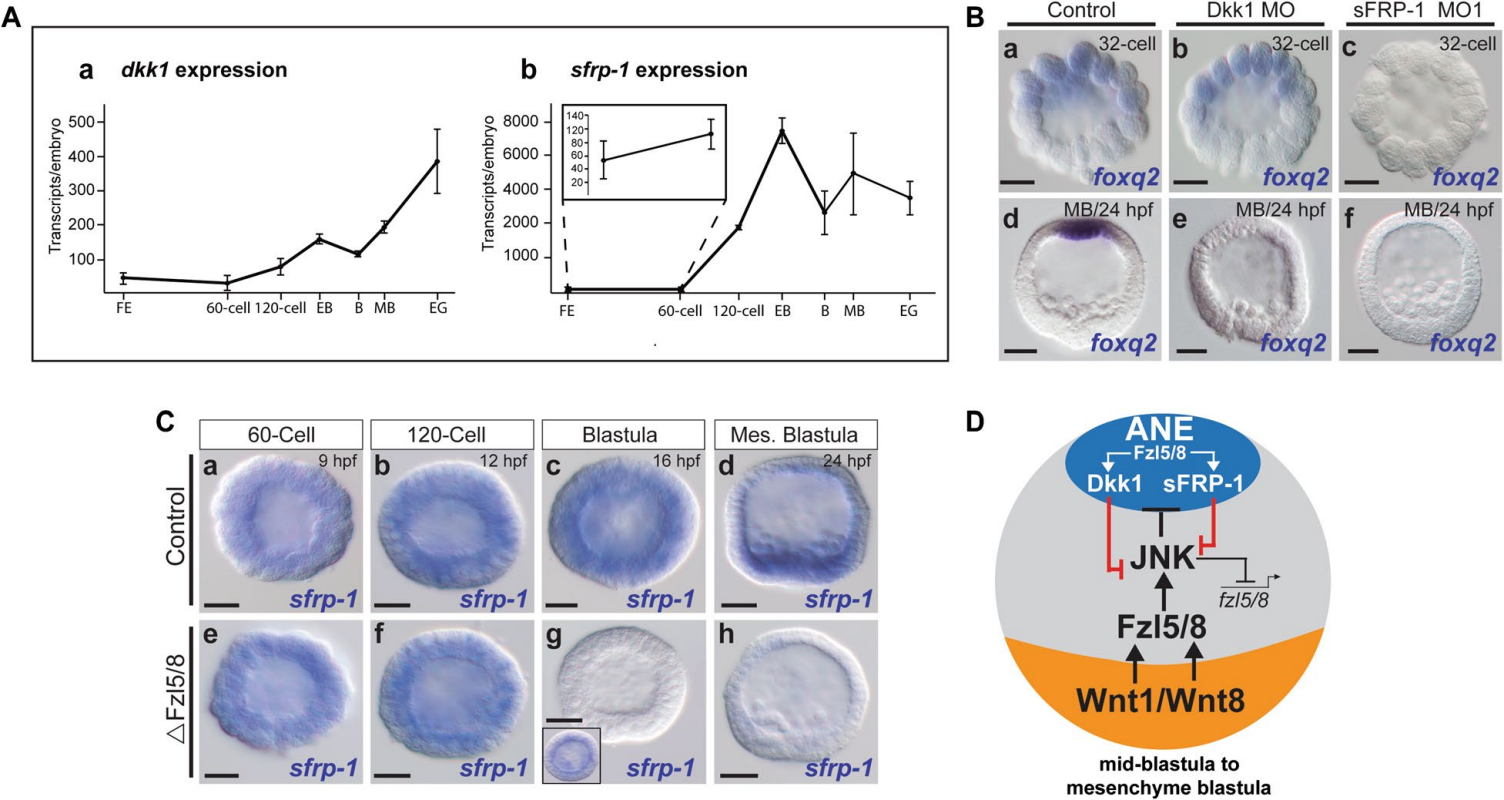
sFRP-1 and Dkk1 antagonize Wnt signaling during final phase of ANE restriction. **A** qPCR measurements showing the temporal expression of *dkk1* (**a**) and *sfrp*-*1* (**b**) transcripts per embryo from egg to mesenchyme blastula stage of development. The inset in **b** shows the number of *sfrp*-*1* transcripts per embryo in fertilized eggs and 60-cell embryos. **B** Comparing *foxq2* expression between Dkk1 morphants (**Bb**, **Be**) and sFRP-1 morphants (**Bc**, **Bf**) during early and late stages of the ANE restriction. **C** Beginning at blastula stages, *sfrp*-*1* is downregulated in embryos in the absence of functional Fzl5/8 signaling (**e**–**h**). **D** A model diagram, showing that both zygotic sfrp-1 and dkk1 are activated by Fzl5/8 signaling in the ANE and that they work together in a negative feedback loop to antagonize Wnt1/Wnt8–Fzl5/8 signaling in the ANE territory. The model is based on the data presented in this and the preceding figures, as well as data from [20]. FE, fertilized egg; EB, early blastula; B, blastula; MB, mesenchyme blastula. MO, morpholino. Scale bar = 20 μm

**Fig. 6:**
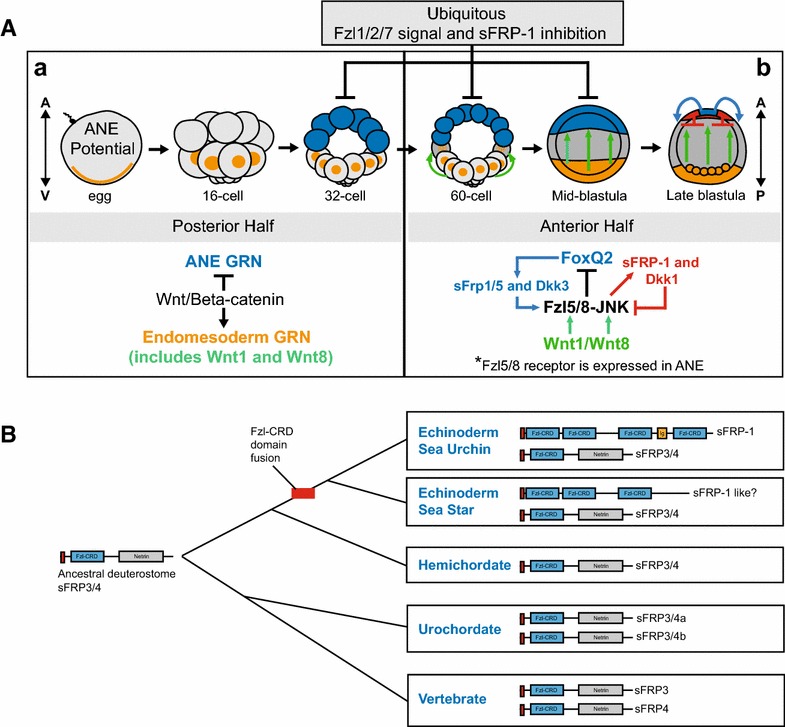
A new model for the Wnt signaling network governing ANE restriction in the sea urchin embryo and the evolution of sFRP3/4 and sFRP-1 in deuterostomes. **Aa** Early downregulation of the ANE GRN from posterior blastomeres. (Posterior half of the embryo) A broad, maternal regulatory mechanism is able to activate the ANE GRN throughout the embryo by the 32-cell stage, but nβ-catenin signaling prevents the expression of the ANE GRN in the posterior half of the 32-cell embryo through an unknown mechanism and activates *Wnt1* and *Wnt8* expression at the 32-cell stage. In addition, around the 32- to 60-cell stage sFRP-1 and Fzl1/2/7 signaling antagonize the downregulation of the ANE GRN in the anterior hemisphere [20]. **Ab** Downregulation of the ANE GRN from cells in the lateral ectoderm domain and the establishment of the ANE around the anterior pole. (Anterior half of the embryo) Around the 32- to 60-cell stage (7–9 hpf), secreted Wnt1 and Wnt8 diffuse into more anterior ectodermal blastomeres (*Wnt8* expression is activated throughout the equitorial ectoderm territory) and signal through the Fzl5/8 receptor, activating the JNK pathway. This Wnt/JNK pathway progressively downregulates ANE GRN expression from the central ectodermal territory during the early to late blastula stages (12–18 hpf). Within the same ectodermal cells, sFRP-1 and Fzl1/2/7 signaling antagonize Wnt1/Wnt8–Fzl5/8-JNK-mediated downregulation of the ANE GRN. Around the mid-blastula stage (16 hpf), an anterior signaling center activated by the cardinal ANE transcriptional regulator FoxQ2 secretes the Wnt modulators Dkk3 and sFRP-1/5 within the regressing ANE territory. Dkk3 and low levels of sFRP-1/5 are necessary to stimulate the Fzl5/8–JNK signaling during the later stages of ANE restriction. During the final phase of the process from early to late blastula stages (18–24 hpf), Fzl5/8 signaling activates the expression of the secreted Wnt antagonists sFRP-1 and Dkk1 in the anterior-most cells around the anterior pole. These antagonists establish a correctly sized ANE territory by preventing the downregulation of ANE factors by antagonizing Fzl5/8 signaling through a negative feedback mechanism [20]. **B** Schematic representation of the expansion of sFRP3/4 paralogues and sFRP-1 during deuterostome evolution

Fzl5/8 signaling activates a negative feedback loop by activating high levels of the secreted Wnt inhibitor Dkk1 around the anterior pole at the end of ANE restriction (late blastula [18 hpf]/mesenchyme blastula stages [24 hpf]). In our previous study, we showed that this high level of Dkk1 blocks Fzl5/8–JNK-mediated downregulation of the ANE GRN and establishes the perimeter of the ANE territory at this stage [20] (see Fig. 6). However, we did not investigate a potential involvement of Dkk1 during early stages of ANE restriction because *dkk1* expression cannot be detected by WMISH until the end of the restriction process at mesenchyme blastula stage (Additional file 3: Figure S3). Low levels of *dkk1* transcripts are detectable in zygotes and through mid-blastula stages by qPCR (Fig. 5Aa) which are similar to the expression levels of *sfrp*-*1* during cleavage stages (Fig. 5b inset). Thus, we hypothesized that these levels of Dkk1 may be sufficient and necessary to antagonize Fzl5/8 signaling during early stages of ANE restriction (32- to 60-cell stage), along with early sFRP-1. To test this idea, we assayed for *foxq2* expression in Dkk1 morphants and sFRP-1 morphants in the same batches of embryos at the very beginning of ANE restriction (late 32-cell stage; 7–8 hpf). As expected, *foxq2* expression was severely downregulated in sFRP-1 knockdown embryos at the late 32-cell stage, but embryos injected with a previously characterized Dkk1 morpholino [20] showed no detectable difference in *foxq2* expression when compared to control embryos (Fig. 5Ba–c). In both cases, *foxq2* was completely eliminated in both sFRP-1 and Dkk1 knockdown embryos by the mesenchyme blastula/early gastrula stage (Fig. 5Bd–f). These results suggest that Dkk1 is only necessary during the later stages of ANE restriction.

The expression pattern of *sfrp*-*1* appears to overlap those of *fzl5/8* and *dkk1* (cf. Additional file 1: Figure S3Ae, Ba, and Bb); thus, we hypothesized that Fzl5/8 signaling activates *sfrp*-*1* in the ANE territory in addition to *dkk1*. To test this hypothesis, we injected zygotes with ΔFzl5/8 mRNA and assayed the expression of *sfrp*-*1* in the embryos at multiple stages during ANE restriction. There was no difference in expression of *sfrp*-*1* between control embryos and ΔFzl5/8 mRNA-injected embryos between the 60-cell and 120-cell stages (Fig. 5Ca, Cb, Ce, Cf). In contrast, expression of *sfrp*-*1* was downregulated in ~ 45% of ΔFzl5/8 mRNA-injected embryos at the hatched blastula stage (16 hpf) (*n* = 73/162) and the percentage of embryos lacking detectable *sfrp*-*1* increased to ~ 94% at the mesenchyme blastula stage (24 hpf) (*n* = 181/193) (Fig. 5Cg, Ch). Taken together, these results suggest that Fzl5/8 signaling is required to activate zygotic *sfrp*-*1* expression beginning midway through ANE restriction, where it acts in parallel with Dkk1 in a negative feedback mechanism to antagonize Fzl5/8 signaling around the anterior pole (Fig. 5D).

## Discussion

We present the first functional characterization of the novel secreted Frizzled-related 1 (sFRP-1) protein, which is expressed during early anterior–posterior neuroectoderm specification and patterning in the sea urchin embryo. Our analyses indicate that maternally supplied sFRP-1 works in concert with, but independently from, the Fzl1/2/7 signaling pathway to antagonize the downregulation of ANE GRN expression by Fzl5/8–JNK signaling during the early stages of ANE restriction (60-cell to blastula stages). Then, Fzl5/8 signaling activates zygotic sFRP-1 around the anterior pole where it works in parallel with Dkk1 in a negative feedback signaling mechanism to antagonize Fzl5/8–JNK signaling during the final phase of ANE restriction, establishing the outer boundary of the ANE territory. Interestingly, sFRP-1 is similar to several poorly characterized genes in many metazoan species that contain one or more well-conserved Wnt binding Fzl-CRD domains [34]. Our study strongly suggests that these Fzl-CRD-containing proteins may be important and possibly novel components of the Wnt signaling mechanisms in the species in which they exist.

We previously showed that there are at least two critical interactions among the Wnt signaling branches during ANE restriction as early as the 32- to 60-cell stages in sea urchin embryos. At this time, Wnt/β-catenin signaling in posterior blastomeres activates the expression of Wnt1 and Wnt8. Our expression and functional data [20] suggest that these ligands diffuse into more anterior ectodermal blastomeres where they activate Fzl5/8–JNK signaling [20], acting as a relay mechanism between Wnt/β-catenin in posterior endomesodermal cells and Wnt/JNK signaling in anterior ectodermal cells (Fig. 6). At the same time, Fzl1/2/7 signaling antagonizes both Wnt/β-catenin and Fzl5/8–JNK signaling, preventing these pathways from being upregulated and completely eliminating the ANE GRN as early as the 32- to 60-cell stage [20]. Here, our data strongly suggest that sFRP-1 activity is necessary from the very beginning to antagonize the activity of Fzl5/8–JNK signaling *only in the anterior half of the embryo* (Fig. 3). The results from these experiments parallel those used to determine the function of Fzl1/2/7 signaling [20], indicating that both maternally supplied mechanisms are critical to moderate Fzl5/8–JNK signaling from the beginning of the restriction process. To date, the exact molecular mechanism(s) by which Fzl1/2/7–PKC signaling antagonizes Fzl5/8–JNK signaling during the early stages of ANE restriction are unknown. It is possible that Fzl1/2/7 signaling antagonizes Fzl5/8–JNK signaling at the intracellular and/or transcriptional level. Alternatively, Fzl1/2/7 signaling could activate a secreted modulator like sFRP-1 that blocks Wnt1/Wnt8–Fzl5/8–JNK signaling at the extracellular level. Our data show that Fzl5/8–JNK signaling activates zygotic *sfrp*-*1,* not Fzl1/2/7 signaling, indicating that sFRP-1 is not a secreted factor activated downstream of Fzl1/2/7 signaling. Instead, we propose that maternally supplied secreted sFRP-1 interferes with Wnt1 and Wnt8 in the extracellular space, preventing the activation of Fzl5/8–JNK signaling in anterior blastomeres together with, but independent from, Fzl1/2/7 signaling during the early stages of the ANE restriction mechanism.

Our data also show that Wnt1/Wnt8–Fzl5/8–JNK signaling must be limited throughout ANE restriction to maintain the steady posterior-to-anterior downregulation of ANE GRN members between the 60-cell and mesenchyme blastula stages [20] (Fig. 6Aa). In previous studies, we have shown that different molecular mechanisms activate two secreted anterior Wnt modulators, sFRP-1/5 and Dkk1 (FoxQ2 activates *sfrp-1/5* and Fzl5/8 signaling activates *dkk1*). Both of these modulators must antagonize Fzl5/8 signaling in anterior ectoderm to maintain the correct rate of ANE GRN downregulation and the establishment of the final ANE territory beginning around the middle of the restriction process (mid-blastula stage) [20, 31]. Here, we show that zygotic *sfrp*-*1* expression is activated by Fzl5/8 signaling around the same time as zygotic *dkk1* and *sfrp-1/5.* Importantly, we show that zygotic sFRP-1 activity is necessary starting around mid-blastula stages to antagonize ANE restriction. Thus, it appears that the negative feedback mechanism activated by Fzl5/8 signaling during these stages activates two secreted Wnt modulators, sFRP-1 and Dkk1, that act in parallel, along with sFRP-1/5, to block Fzl5/8 signaling around the anterior pole and establish the final ANE territory (Fig. 6Ab pathway in red). Interestingly, both of these secreted modulators are essential to prevent downregulation of the ANE GRN because knocking down either one individually allows ANE downregulation to continue to the anterior pole. Together, these data are consistent with the idea that the early role of both maternal sFRP-1 and Fzl1/2/7 signaling is to separately act to moderate the activity of Fzl5/8–JNK signaling so that the wave of posterior-to-anterior downregulation of ANE GRN expression occurs at a specific rate. Then, in a coordinated spatiotemporal transition during the middle of ANE restriction, the cardinal transcriptional regulator FoxQ2 and Fzl5/8 signaling turn on the zygotic expression of three secreted Wnt modulators whose separate, but combined activity, in addition to the possible negative input of Fzl1/2/7 signaling, is able to build to a level that is capable of stopping the ANE restriction mechanism and defining the outer boundary of the ANE territory.

The eumetazoan ancestor appears to have possessed a set of Frizzled-CRD containing genes including four receptors (Fzl1/2/7, Fzl5/8, Fzl9/10, and Fzl4) and two secreted Wnt signaling modulators (sFRP-1/2/5 and sFRP3/4). This core ancestral gene group is thought to have been maintained in many invertebrate metazoan species until whole genome duplications increased the number of orthologues in vertebrates [33, 34]. The unique domain architecture of protein families has traditionally been considered to be a result of duplication and modifications from a common ancestral gene [45]. Consistent with this idea, the vertebrate sFRP3/4s, urochordate sFRP3/4a and b, and the sea urchin sFRP3/4 maintained the domain organization of the original/ancestral sFRP3/4. sFRP-1 appears to be related to ancestral sFRP3/4 s [35]; however, the domain organization of sFRP-1 is novel, containing 4 tandemly arrayed Fzl-CRD domains and an Ig domain instead of one Fzl-CRD and Netrin domain. Interestingly, recent phylogenetic studies indicate that it is not uncommon for several novel Fzl-CRD-containing genes to exist in various taxa in addition to the more conserved core group of Fzl-CRD containing genes [46, 47]. Similar to the domain structure of sFRP-1, many of the novel proteins encoded by these genes are predicted to be secreted and they often contain one or more Fzl-CRD domains related to the Frizzled receptors or sFRPs [34, 48]. These data and others led Pei and Grishin to propose that Fzl-CRD domains may act as “mobile evolutionary units” that undergo domain fusion events, bringing new genes into Wnt signaling in the species in which they exist [48]. Given the unique domain architecture of sea urchin sFRP-1, it is thus possible that one or more of these mobile Fzl-CRD evolutionary units may have been involved in a domain fusion event(s) that lead to a novel gene related to the ancestral sFRP3/4 (Fig. 6B). Interestingly, the echinoderm sea star genome contains at least four genes that are related to sFRP-1 and possess a similar domain architecture (Fig. 6B), whereas hemichordates, which from a sister phylum with echinoderms, appear to lack any such gene. Together, these data suggest that sFRP-1 may have emerged after the divergence of echinoderms and hemichordates and then become integrated into the Wnt signaling network governing AP patterning in the sea urchin embryo.

Several possible outcomes can arise once a new functional gene appears in a genome. For example, if the new gene emerges from a duplication, it could play redundant functions as the paralogue, leading to new morphologies if spatiotemporal expression is different from the original gene.

The new gene could also take over the role of the ancestral gene, allowing the ancestral gene to take on a new role in a different territory and/or at different developmental stages [1, 5, 49]. We find it interesting that the ancestral sea urchin s*frp3/4* gene is not expressed during early embryonic AP specification and patterning given the prominent role the sFRP3/4 orthologue FrzB plays during AP neuroectoderm patterning in vertebrate deuterostomes. In *Xenopus* and zebrafish embryos, FrzB is expressed in the ANE territory (anterior pre-chordal plate) where it is necessary to antagonize posterior-to-anterior Wnt signaling and help establish the territory that will form the forebrain and eye field [37–39]. While there are no functional studies on sFRP3/4 outside of the vertebrates, *sfrp3/4* is expressed in anterior regions of hemichordate embryos during gastrula stages, as in vertebrates (Chris Lowe and Sebastien Darras labs, personal communication), suggesting that this ancestral sFRP3/4 may play a role in early AP neuroectoderm patterning in these organisms. Thus, it is tempting to speculate that sFRP-1 may have assumed the ancestral role of sFRP3/4 in the sea urchin embryo and that other deuterostome embryos may have retained it.

## Conclusion

We have a limited understanding of how the different Wnt signaling branches involved in Wnt networks interact at the extracellular, the intracellular, or transcriptional levels to control one another’s activity in an in vivo developmental context. In this study, we have shown that the novel sea urchin protein, sFRP-1, is a critical member of a growing list of extracellular Wnt modulators, including Dkk1, Dkk3, and sFRP-1/5, that precisely control how information from the Wnt/β-catenin, Wnt/JNK, and Wnt/Ca^2 +^ pathways is integrated during specification and patterning of territories along the AP axis in sea urchin embryos. Given the relative lack of attention paid to the functional role of novel genes in development, our results also provide an important illustration of how these genes can become key players in the otherwise highly conserved signaling pathways that govern fundamental developmental mechanisms.

## Methods

### Embryo cultures

*Strongylocentrotus purpuratus* were obtained from Monterey Abalone Company, Monterey, CA, and Point Loma Marine Invertebrate Lab, Lakeside, CA. The adult sea urchins were injected with 0.5 M KCL into their body cavities in order to collect the eggs and sperm. The eggs were washed two times with artificial sea water (ASW) and then fertilized in a glass beaker or a plastic culture dish by adding a 1:1000 dilution of sperm. Once fertilized, embryos were cultured in artificial seawater (ASW) at 15 °C.

### mRNA and morpholino injections

Double-stranded DNA was synthesized (Integrated DNA Technologies) based on the published full-length sequence [43] and the sea urchin genome sequence [44]. The full-length *sfrp*-*1* clone was inserted into pCS2 + vector using the following primers: Forward 5′-GCGATGGAGTTTCCACCTCA-3′; Reverse 5′-GAAGACTCACACAGCTCCCG-3′. sFRP-l-pCS2 was linearized with NotI, and mRNA was synthesized using the Sp6 mMessage mMachine Kit (Ambion). Overexpression studies were performed by injecting ~ 20 pL of full-length *sfrp*-*1* (2–3 μg/μL) and *dkk1* (3 μg/μL) as well as *ΔFzl5/8* (2.0 μg/μL) mRNA into zygotes.

For loss-of-function experiments, two different morpholino-substituted oligonucleotides were designed from *S. purpuratus* EST and genomic sequences [43]. A splice-blocking morpholino was designed to target the first exon–intron boundary of *sfrp*-*1*, resulting in a transcript lacking sequence from the second exon. The morpholinos were produced by Gene Tools LLC (Eugene, OR). The sequences and injection concentrations were as follows: sFRP-1 MO1 (translation blocking): 5′-CGCTGTGACAGGTGTTCTCTTCGAT-3′ (0.75–0.85 mM); sFRP-1 MO2 (splice blocking): 5′-CGGAAGATATTATAGGCATACCTGT-3′ (2.25–2.5 mM); Dkk1 MO1: 5′-GCGTCTAAATCCTAAATTCCTTCCT-3′ (1.5–1.6 mM) [20].

For microinjections, eggs were de-jellied by passing them through 74 μm mesh Nitex. The eggs were arrayed in rows on a plastic culture dish coated with 25% protamine sulfate and fertilized by addition of diluted sperm. Immediately after fertilization, embryos were injected with a solution containing 15% FITC (2.5 μg/mL), 20% glycerol, and morpholino oligonucleotides and embryos were cultured at 15° C. Microinjection experiments were performed using at least three different batches of embryos, consisting of 30–200 embryos in each experiment. Experiments were considered conclusive only if a change in phenotype or marker expression was observed in at least 85% of the injected embryos.

### Whole-mount in situ hybridization

Probes for each gene analyzed correspond to the full-length cDNA sequence. In situ hybridization and detection by alkaline phosphatase staining were carried out as previously described [19, 20, 50].

### Quantitative polymerase chain reaction (qPCR)

qPCR experiments were performed as described previously [19]. For each experiment, embryos from three different mating pairs were used and each reaction was carried out in triplicate. The primers used for qPCR reaction were: sFRP-1, Forward-5′CGAGACGACTATTGCAGATG3′; Reverse- 5′ATTCCTCAGGGGAGTTAGG3′ and Dkk1, Forward-5′GTGTTCGCAAGGTCTCTC3′ and Reverse-5′GTCGTTCTTGCTCGGAAG3′. Primer set information for the rest of the ANE GRN genes is provided in [20]. ΔCt was used to calculate expression level for *sfrp*-*1* relative to *z12* in real-time quantitative PCR experiments. To determine the expression levels, the numbers of transcripts per embryo were estimated based on the ΔCt value of *z12* transcript [51]. In differential expression comparisons between control and perturbed embryos mitochondrial 12 s RNA Ct values were used to normalize the relative concentration of mRNA; a twofold or higher change in gene expression level was considered to be significant.

### Phylogenetic analysis

For the phylogenetic analysis, we used truncated amino acid sequences of the Fzl-CRD domains from each protein (Additional file 4: Table S1). The extracted sequences were aligned using Muscle [52] and manually edited with BioEdit [53]. Phylogenetic relationships among them were estimated using maximum likelihood and Bayesian approaches. We run maximum likelihood analyses in IQ-Tree ver 1.5.5 [54] as implemented in the IQ-Tree web server [55] last accessed on June 2017 and evaluated support for the nodes with the ultrafast bootstrap method [56]. We run Bayesian analyses in MrBayes version 3.2 [57], setting four simultaneous chains for 2 × 10^7^ generations, sampling trees every 1000 generations, and using default priors. We assessed convergence by measuring the standard deviation of the split frequency among parallel chains. We summarized results with a majority-rule consensus of trees collected after convergence was reached.

## Additional files

**Additional file 1: Figure S1.** Phylogenetic analysis of sea urchin Frizzled-like cysteine-rich domains and *sfrp3/4* expression during ANE restriction. **(A)** Fzl-like cysteine-rich domain maximum likelihood analysis. The Bayesian tree was built using only Fzl-CRD amino acid sequences. Each Fzl-CRD orthologue is highlighted by a different color. Those proteins that contain more than one Fzl-CRD are labeled with a numbered CRD corresponding to its position in the protein. **(B)** qPCR measurements from three different cultures of embryos showing the approximate number of *sfrp3/4* transcripts per embryo at 0 hpf, 60-cell, 120-cell, hatched blastula, and mesenchyme blastula stages. The *y* axis shows the approximate number of transcripts per embryo based on the Ct value of *z12* transcripts. The absolute concentrations of *z12* transcripts are known at each stage [60]. FE, fertilized egg; EB, early blastula; B, blastula; MB, mesenchyme blastula.

**Additional file 2: Figure S2.** sFRP-1 morpholino control experiments. (A) The expression of ANE regulatory factors *six3*, *foxq2, nkx3.2*, and *sfrp-1/5* are severely downregulated in embryos injected with sFRP-1 morpholino 2 designed to interfere with the splicing at the first exon–intron boundary. (B) Low levels of sFRP-1 expression (1 μg/μL) rescue embryos injected with sFRP-1. MO1. The number of embryos examined that show the representative phenotypes depicted is indicated in each panel. sFRP-1 MO1 does not bind to exogenous *sfrp*-*1* mRNA. (C) Diagram of the intron–exon organization of *sfrp*-*1* pre-mRNA. Primers used to characterize the mRNA products (orange arrows). Position of the target sequence for the morpholino (red bar). MO, morpholino. (D) Efficacy control for the sFRP-1 splice-blocking morpholino. PCR analysis of control glycerol injected and embryos injected with a sFRP-1 splice-blocking morpholino (sFRP-1 MO2 in methods**)**. Expected control PCR product size for *sfrp*-*1* = 100 bps; no PCR product expected from sFRP-1 MO2-injected embryos.

**Additional file 3: Figure S3.** Spatiotemporal expression analysis of Dkk1, Fzl5/8, and sFRP-1. (A) Whole-mount in situ hybridization analysis of *dkk1* expression during ANE restriction. (B) *sfrp*-*1* (a) and *fzl5/8* (b) mRNA transcripts are expressed in remarkably similar territories mesenchyme blastula stage embryos (24 hpf).

**Additional file 4: Table S1.** Frizzled-like cysteine-rich domains used for phylogenetic analysis.
