# Supplementary material for: A novel gene’s role in an ancient mechanism: secreted Frizzled-related protein 1 is a critical component in the anterior–posterior Wnt signaling network that governs the establishment of the anterior neuroectoderm in sea urchin embryos
Source: EvoDevo. 2018 Jan 22;9:1. doi: 10.1186/s13227-017-0089-3 (PMC5778778; doi:10.1186/s13227-017-0089-3)

Supplementary Figure 1. Phylogenetic analysis of Fzl-CRD contaning proteins and the expression of *sfrp3/4* during ANE restriction in *S. purpuratus* embryos.

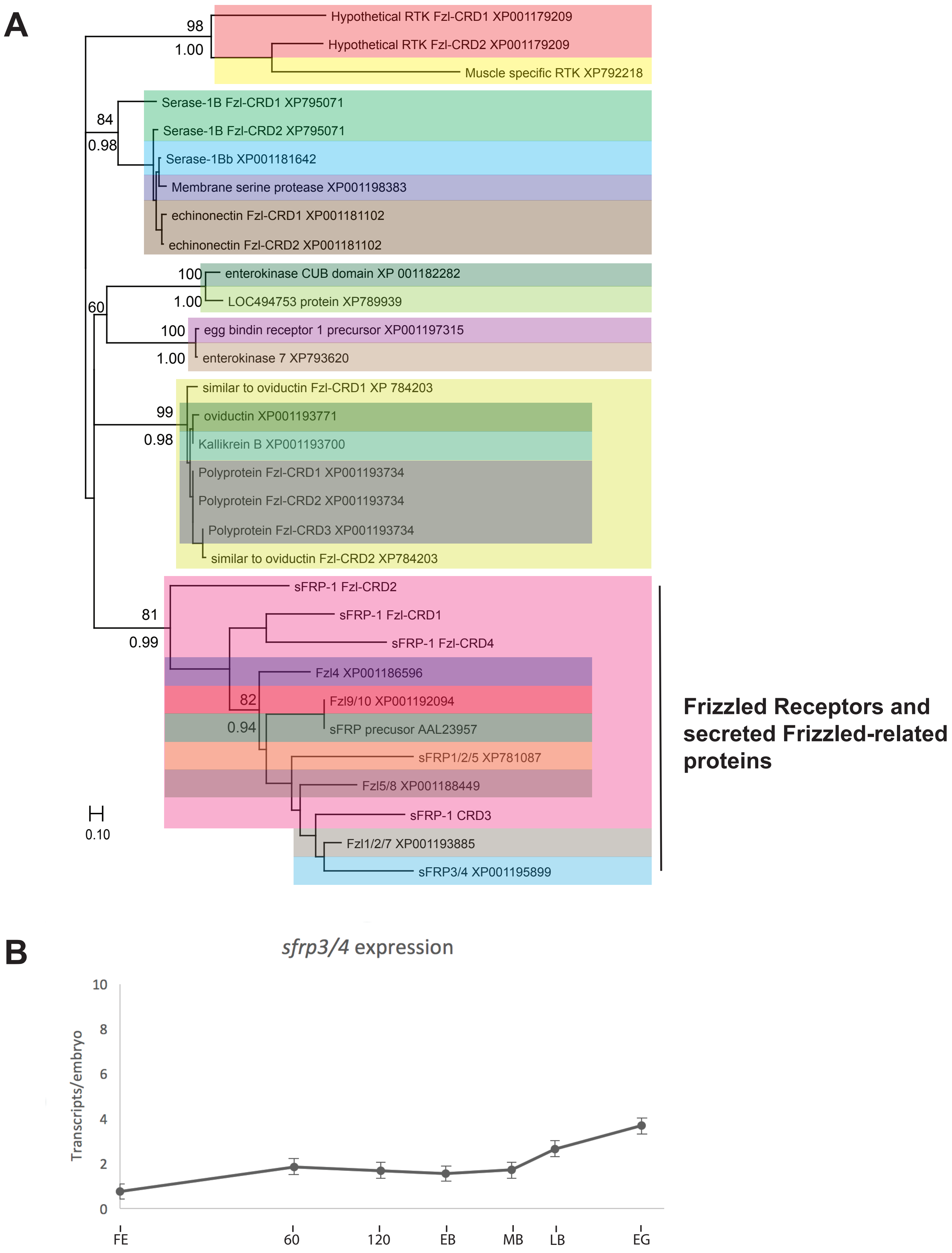

Supplement: Supplementary file 1 — Additional file 1: Figure S1. Phylogenetic analysis of sea urchin Frizzled-like cysteine-rich domains and sfrp3/4 expression during ANE restriction. (A) Fzl-like cysteine-rich domain maximum likelihood analysis. The Bayesian tree was built using only Fzl-CRD amino acid sequences. Each Fzl-CRD orthologue is highlighted by a different color. Those proteins that contain more than one Fzl-CRD are labeled with a numbered CRD corresponding to its position in the protein. (B) qPCR measurements from three different cultures of embryos showing the approximate number of sfrp3/4 transcripts per embryo at 0 hpf, 60-cell, 120-cell, hatched blastula, and mesenchyme blastula stages. The y axis shows the approximate number of transcripts per embryo based on the Ct value of z12 transcripts. The absolute concentrations of z12 transcripts are known at each stage [60]. FE, fertilized egg; EB, early blastula; B, blastula; MB, mesenchyme blastula. [file 13227_2017_89_MOESM1_ESM.pdf]
