# Supplementary figures and images for: A novel gene’s role in an ancient mechanism: secreted Frizzled-related protein 1 is a critical component in the anterior–posterior Wnt signaling network that governs the establishment of the anterior neuroectoderm in sea urchin embryos
Source: EvoDevo. 2018 Jan 22;9:1. doi: 10.1186/s13227-017-0089-3 (PMC5778778; doi:10.1186/s13227-017-0089-3)

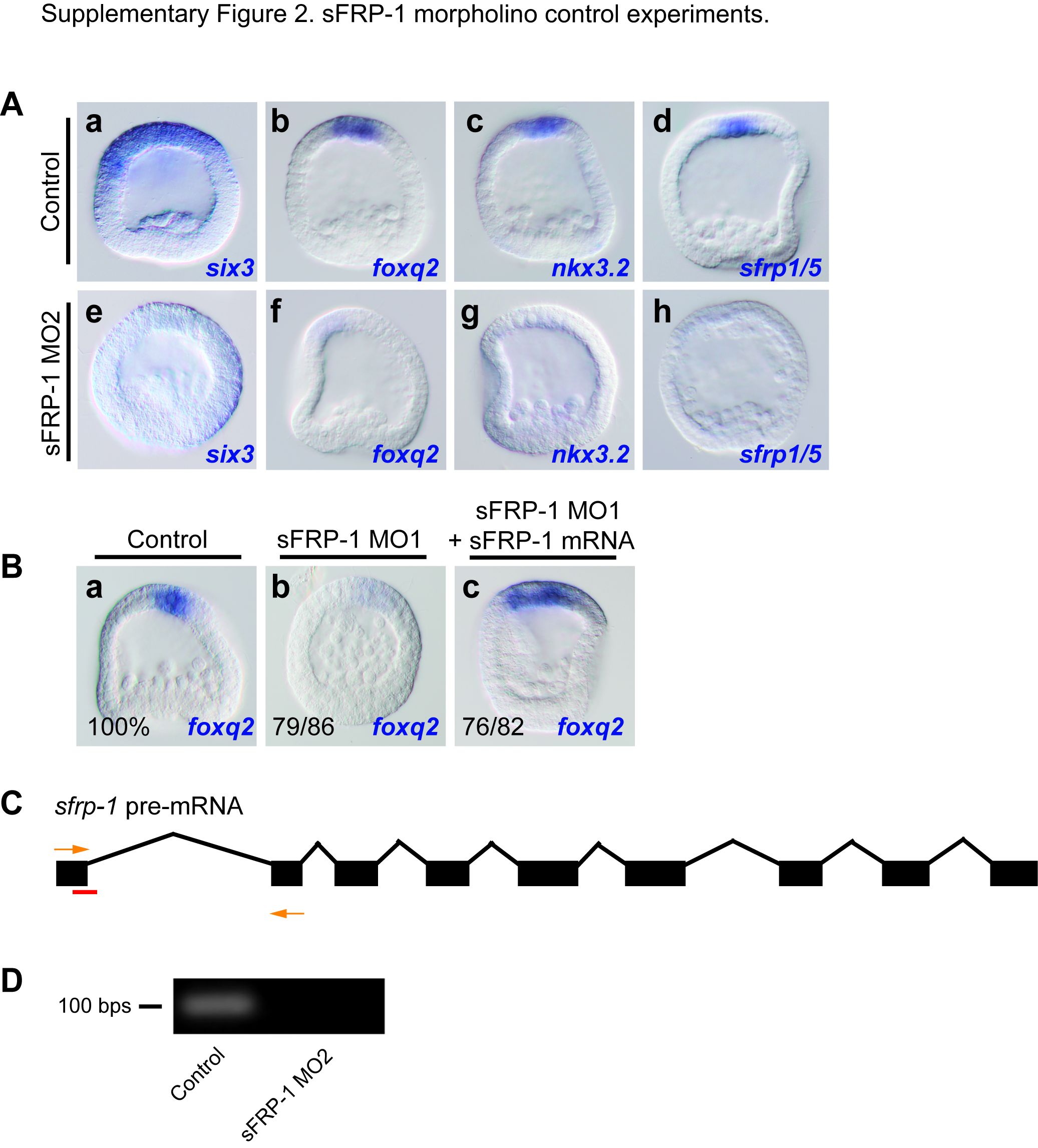

Supplement: Supplementary file 2 — Additional file 2: Figure S2. sFRP-1 morpholino control experiments. (A) The expression of ANE regulatory factors six3, foxq2, nkx3.2, and sfrp-1/5 are severely downregulated in embryos injected with sFRP-1 morpholino 2 designed to interfere with the splicing at the first exon–intron boundary. (B) Low levels of sFRP-1 expression (1 μg/μL) rescue embryos injected with sFRP-1. MO1. The number of embryos examined that show the representative phenotypes depicted is indicated in each panel. sFRP-1 MO1 does not bind to exogenous sfrp-1 mRNA. (C) Diagram of the intron–exon organization of sfrp-1 pre-mRNA. Primers used to characterize the mRNA products (orange arrows). Position of the target sequence for the morpholino (red bar). MO, morpholino. (D) Efficacy control for the sFRP-1 splice-blocking morpholino. PCR analysis of control glycerol injected and embryos injected with a sFRP-1 splice-blocking morpholino (sFRP-1 MO2 in methods). Expected control PCR product size for sfrp-1 = 100 bps; no PCR product expected from sFRP-1 MO2-injected embryos. [file 13227_2017_89_MOESM2_ESM.tif]

Supplementary figure 3. Spatiotemporal expression of Dkk1, Fzl5/8 and sFRP-1 during ANE restriction.

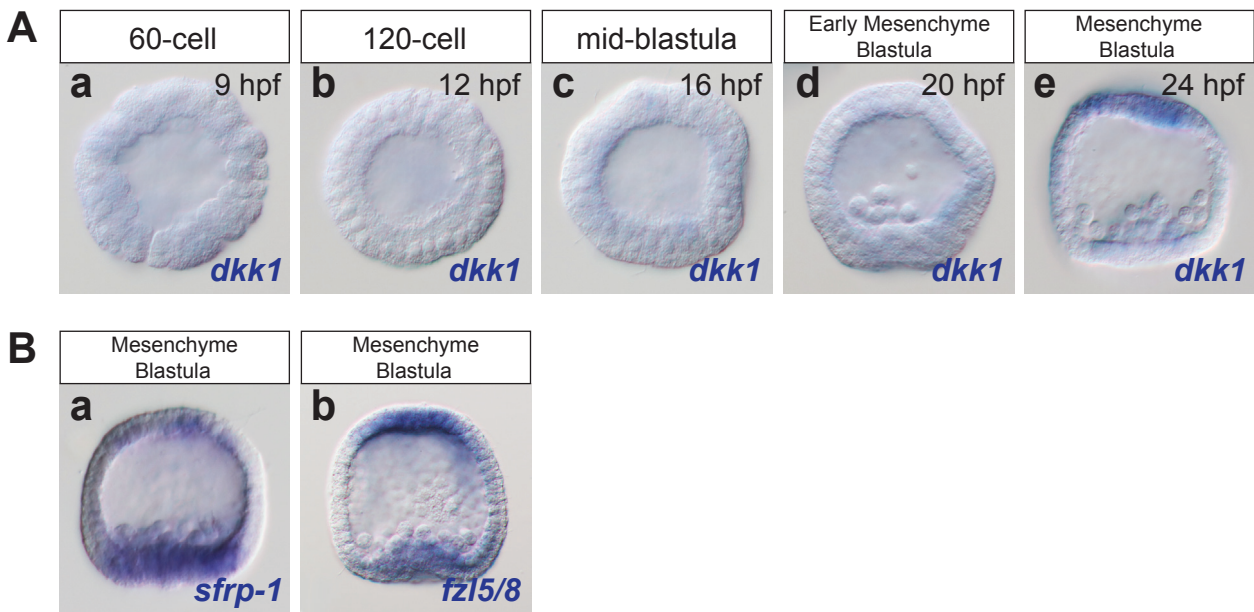

Supplement: Supplementary file 3 — Additional file 3: Figure S3. Spatiotemporal expression analysis of Dkk1, Fzl5/8, and sFRP-1. (A) Whole-mount in situ hybridization analysis of dkk1 expression during ANE restriction. (B) sfrp-1 (a) and fzl5/8 (b) mRNA transcripts are expressed in remarkably similar territories mesenchyme blastula stage embryos (24 hpf). [file 13227_2017_89_MOESM3_ESM.pdf]
