# Supplementary material for: A novel gene’s role in an ancient mechanism: secreted Frizzled-related protein 1 is a critical component in the anterior–posterior Wnt signaling network that governs the establishment of the anterior neuroectoderm in sea urchin embryos
Source: EvoDevo. 2018 Jan 22;9:1. doi: 10.1186/s13227-017-0089-3 (PMC5778778; doi:10.1186/s13227-017-0089-3)
Supplement: Supplementary file 4 — Additional file 4: Table S1. Frizzled-like cysteine-rich domains used for phylogenetic analysis. [file 13227_2017_89_MOESM4_ESM.pdf]

Supplementary Table 1

sFRP-1 Fzl-CRDs AF426109:

sFRP-1 CRD1
CEPLTMPLCLQNGVQYTETIFPNLAGMMTQQDAIVASESLAPLVAIGCGPIVDFVCTLFAPPCLEVGDVSV
PVPPCRHVCEEARDQCMPIFQTVGMPWPDQFECSRFPAIATEGPARCAEP

sFRP-1 CRD2
CYDVTVPECRQMGFTHTKFPNIFGHTTPEEMAQDMTSLTGLILSGCSDDLLPLICAA
YLPKCDSETGDVTKPCKETCRRISKDCKDAMKELSIGRLDIFTCRNYPSKKDGECVEDV

sFRP-1 CRD3
CESVSTPICMDMKYDFTQFPNLLQHANQDVAYLELSQFAPLIQVECSLDLGAFLCAVYMPPCTGQSSPQ
GIKPPCRELCESSRVGCLPLMTKFGYTWPETLDCDQFPLGSEVGKNCFSNE

sFRP-1 CRD4
CEALESAQCDMVLPYSASLYPNSMGHMTPTEAELASFEFLPLLAMGCSPVNLPLFVCSLHFPSCGDAPV
LPCSELCRAAMTECSVFILGIQHVWPEKAQCSNFPSMAEGTCIPP

Fzl910 XP001192094
GHCERITIDFCQGIGYNMTKMPNLFGHVTQSEAAPSIHEFHPLVVVGCAEHLRLFLCSMYAPMCSEHIDIRI
PSCQSMCDDVRRKCEPVMARFSFSWPESMNCDKLPRGKDLCMVAPD

Fzl4 XP001186596
TVMCEPITIKLCQGVGYNMTRMPNLVGHELQQDAELQLQTFTPLIQYGCSPFLRFFLCSVYAPYCTDKIDV
PISVCRPLCERVRASCNPVLSKFGFKWAEALNCSQFLERSDGNDICMGPPPGEGD

Fzl1/2/7 XP001193885
GRCEAITIPLCEDMPYNMTIMPNLLNHQHQEDAGLEVHQFYPLVKVQCSEDLKNFLCSMYAPVCTVLDSA
LPPCKSLCLSARNGCESLMNKFGFSWPASLSCDQFPEGGLCFDRNR

Fzl5/8 XP001188449
KCEAITVPMCRDVGYNMTYMPNHFNHDSQEEAGLEVHQFYPLVEIQCSPDLKFFLCSMYTPICLADYHKP
LMACRSVCERAKAGCAPLMRQYGFAWPDRMQCENLPEFGDPDNLCMDSNH

sFRP1/2/5 XP781087
PKETDCVDIPEDLSLCQNIGYTRMRMPNLLEHDSLDEVRNQAKSWVPLLLQRCHEGTQLFLCTLFAPVCL
EVDRPIYPCRSLCEEVQAGCGPLMESNCFPWPAMLQCDKFPEDNDLCIRLNMRNQNGT

sFRP3/4 XP001195899
RPECQLVQLPMCESMPYNLTRMPNLLHHSTQENAKLAIEQFLPLVNTNCSGNLTFFLCAMYVPICTMGFM
EEPVPPCKSVCQNVRAGCEPIMIEHNVSWPVYLSCDQLPEYTSGVCITRDAIV

Enterokinase CUB domain XP001182282
TCQPVPSECSSVLPWTTTVFPNARYQSESDALQRYAELESTALSCDPSGQLLLCAALFPECPHYGTTRN
LCPSVCEAVIDNCP

sFRP precursor AAL23957
CERITIDFCQGIGYNMTKMPNLFGHVTQSEAAPSIHE\FHPLVVVGCAEHLRLFLCSMYAPMCSEHIDIRIP
SCQSMCDDVRRKCEPVMARFSFSWPESMNCDKLPRGKDLCMVAPD

Serase-1B XP001181642
CEQVQFDICRQNLAYNLTFFPNRNAETADAAAGSFTDMADTISSCHDHLFPIMCSVYYPECTH
NGPTHRVCYSDCLAVTDACKASFEQLLDRPWPVNCSMFTDE

Egg bindin receptor 1 precursor XP001197315
RCEPIQEGSCLGLLPFNQTYYPSSLADINQTLAIESFSVLSAAATTCHTDASLFLCAVLFPECIHDGPTARP
CQTTCEAVRSACSSAFESLTGSPWAIACE

Oviductin XP001193771
QCTDLRFSTCEEMLSYDRTYFPNPTAQDRDSAISLIEETSILEECHSDFLLLFCNMLFADCPHGGPSRRPC
KALCEEVTDACRESYKALMDKDWPIDCRQLSDDENHEESYCMGGEG

Serase-1B Fzl-CRDs XP795071:

Serase-1B CRD 1
CEPVQLEVCRERLAYNTTLFPHRLAQNAQDAERNFTEIVSSISSCHENLLSLTCNLLYPECTHNGPAQRV
CYSDCVAITDACQDPFEQSLNRPWPFDCSQLTDDYAGEGLCFAAEG

Serase-1B CRD 2
CEQVQFDICRQNLAYNLTFFPNRNAETADAAAGNFTEIEAAISSCHDHLFPFMCSVYYPECTHNGPTHRL
CYSDCLAVTDACKASFEQLLDRPWPVNCSTLNDEQEEDGSCFGPAG

LOC494753 protein XP789939
TCQPVPSVCSSRLAWTTTVFPNARYHTESDALQGYADLEFIALPSCGESALVLICAALFPECPHYGTTINL
CPSVCEAVIDDCPMLSE

Membrane serine protease XP001198383
CEQVHLDICRQSLAYNLTFFPNRNAETADAAAGGFKDMADTISSCHDHLFPFMCTVYYPECTHNGPTHR
VCYSDCLAVTDACKASFEQLLDRPWPVNCSMFTDEQEEDGSCFGPAG

Hypo RTK Fzl-CRDs XP001179209:

Hypo RTK CRD1
PQCEPYRGSVCSPHIANSQVYIPAGQTQADIEAALGAAIQQINNDLPITLNQRCTKYLKPSMCLTAFPLCR
ERPRLAVHRMCYDECRLLTTEICSSLVDYVDAQPELGLVDMLPICTDLPLPGAVAGANSENSCLRMGMS

Hypo RTK CRD2
GYCQPYRGMTCSNFISNHSIYVTDFQAQKLIDERLTQAFLLMARDLSPQCQQYAIPSLCFFAFPFCDETR
QEPRGRELCRDECEILEQDICKTEYQIAKEMPNVILPDCSRLPAIGTNANANCIRVGLP

Muscle specific RTK XP792218
YCAAYNGATCHNQLPSSPYYIFVNGHGDEPLALDNKLSEILSSKTMTSMPEDCQAGIRELLCHATHP
KCQVVGQKPRGQKICKEDCEIVQSMCPEWAQLVNRFLDPLPDKIRSLGLSSCENLPENDCTSL

Enterokinase7 XP793620
RCEPIKESSCLGLLPYNQTYYPSSLADINQTLAIESFSVLSAAATTCHTDASLFLCAVLFPECIHDGPTARP
CQTTCDAVRSACSSAFESLTGSPWAIACE

Kallikrein B XP001193700
HCTDLRFSTCEEVLSYDRTYFPNPTAQDRDSAISLIEETSILEECHEDFLLLFCSMLFADCPHGGPSRRPC
KALCEEVTDACRESYKALMDEDWPIDCRQLSDDENLEESYCMGGEG

Echinonectin Fzl-CRDs XP001181102

Echinonectin CRD1
CEQVQFDKCRQNLAYSVTFFPNGNAETADAAAESFTEIEATISSCHDHLIPFMCSVYYPECTHNGPTHRV
CYSDCLAVTDACKASFEQLLDPPWPVNCSMFTDEQEEDGSCFGPAG

Echinonectin CRD2
CEQVQFDICRQNLAYSVTFFPNGNAETADAAAESFTEIADTISSCHDHLFPFMCSVYYPECTHNGPTHRV
CYSDCLAVSDACKASFEQLLDRPWPVNCSMFTDEQEKDGSCFGPAG

Polyprotein Fzl-CRDs XP001193734:

Polyprotein CRD1
CTDLRFSTCEEVLSYDRTYFPNPTAQDRDSAISLIEETSILEECHEDFLLLFCSMLFADCPHGGPSRRPCK
ALCEEVTDACRESYKALMDEDWPIDCRQLSDD

Polyprotein CRD2
CTDLRFSTCEEVLSYDRTYFPNPTAQDRDSAISLIEETSILEECHEDFLLLFCSMLFADCPHGGPSRRPCK
ALCEEVTDACRESYKALMDEDWPIDCRQLSDD

Polyprotein CRD3
CTDFRFSTCEEVLSYDRTYFPNPTAVDRDSAISLIEETSILEECHENFLLLFCSMLFADCPHGGPSRRPCK
ALCEEVTDACRGSYKELINEDWPIDCRQLSDD

Similar to oviductin Fzl-CRDs XP784203.2:

similar to oviductin CRD1
QCTDLRFSTCEEVLSYNRTYFPNPTAQDRDSAISLIENTSILEECHEDFLLLFCGMLFADCPHGGPSRRPC
KALCEEVTDACRESYLALVDAKWPIDCRHLSNEGNLEESYCIGGEG

similar to oviductin CRD2
QCTDFRFSTCEEVLSYDRTYFPNPTAVDRDSAISLIEETSILEECHENFLLLFCSMLFADCPHGGPSRRPC
KALCEEVTDACRGSYKELINEDWPIDCRQLSDDGNIDQSYCIGGEG
